# Postural Analysis in Temporomandibular Joint Dysfunction (TMJD) Patients: Correlation With Head and Neck Positioning

**DOI:** 10.1155/ijod/6783876

**Published:** 2026-06-17

**Authors:** Amir Jalal Abbasi, Abbas Karimi, Mahsa Sadat Seyedi Konjani, Shamim Chinian, Mona Mohajeri Tehrani

**Affiliations:** ^1^ Department of Oral and Maxillofacial Surgery, Sina Hospital, Tehran University of Medical Sciences, Tehran, Iran, tums.ac.ir; ^2^ Department of Oral and Maxillofacial Surgery, Dental School, Tehran University of Medical Sciences, Tehran, Iran, tums.ac.ir; ^3^ Craniomaxillofacial Research Center, Shariati Hospital, Tehran University of Medical Sciences, Tehran, Iran, tums.ac.ir; ^4^ Department of Oral and Maxillofacial Surgery, Shariati Hospital, Tehran University of Medical Sciences, Tehran, Iran, tums.ac.ir; ^5^ Tehran University of Medical Sciences, Tehran, Iran, tums.ac.ir

**Keywords:** bruxism, clenching, craniohorizontal angle, craniovertebral angle, forward head posture, posture, temporomandibular joint disorders

## Abstract

**Background:**

The relationship between head–neck posture and temporomandibular joint dysfunction (TMJD) remains a topic of controversy, with existing studies reporting inconsistent findings. This study aimed to investigate this relationship by comparing specific postural angles in TMJD patients and healthy controls.

**Materials and Methods:**

A case–control study included 65 TMJD patients and 65 healthy controls (aged 18–50), analyzing head positioning angles (craniohorizontal angle [CHA] and craniovertebral Angle [CVA]) using anatomical markers in photographs processed with Adobe Photoshop. Data were analyzed using SPSS, employing *t*‐tests, chi‐square tests, and multiple linear regression.

**Results:**

The final analyzed sample consisted of 43 TMJD patients and 40 controls after applying inclusion/exclusion criteria and ensuring data completeness. The TMJD group exhibited significantly lower mean CVA (41.1° ± 6.61°) compared to controls (56.83° ± 4.33°; *p* = 0.027), indicating a more forward head posture. The difference in CHA was not statistically significant. Regression analysis identified that a lower CVA (i.e., worse posture) was significantly associated with older age (*β* = −0.45, *p*  < 0.01), bruxism (*β* = −0.32, *p*  < 0.01), and clenching (*β* = −0.28, *p*  < 0.05). These factors explained 77.8% of the variance in CVA (*R* ^2^ = 0.778).

**Conclusion:**

These findings suggest that postural assessment of the CVA and the clinical management of parafunctional habits like bruxism and clenching should be integrated into the comprehensive evaluation and treatment of patients with TMJD.

## 1. Introduction

The temporomandibular joint (TMJ) is one of the critical joints in the body, facilitating mandibular movements. Its articular surface is covered with fibrocartilage, with a high regenerative capacity. The temporalis and masseter muscles are responsible for the majority of this joint’s movements [1].

The term TMJD (TMJ dysfunction [or disorder]) includes several clinical problems involving the TMJ, the masticatory muscle system, or both. Patients suffering from these disorders may experience the following symptoms: facial pain in the areas surrounding the TMJ or masticatory muscles, limitation or deviation in the range of movements of the lower jaw, or the sounds produced during TMJ movement, such as clicking [2]. Chronic pain in TMJD may also be associated with headache, neck pain, and shoulder pain [3]. The global pooled prevalence of any TMJD is quite common, affecting 31.7% (95% CI: 26.3%–37.4%) global population, especially women 20–40 years of age, who suffer from it (significantly higher in females (38.7%) compared to males (22.4%)) [2, 4]. Although TMJD is considered a multifactorial disease, these five etiologies account for the majority of cases: occlusion, trauma, deep pain stimulus, parafunctional activities, and physiological factors such as anxiety and depression [5, 6]. Two hypotheses of lack of occlusal coordination and psychological disorders have been proposed in scientific literature.

Anatomical correlations between the craniomandibular complex and the cervical spine have been evaluated clinically; however, data on correlations between head and neck position are inconsistent. Prior studies on TMJD and head position, considering craniocervical changes, mandibular function, and occlusion, show mixed results [7–9]. Some articles report an absence of a correlation, whereas others acknowledge its existence. Recent evidence further supports a functional link between postural deficits, pain, and TMJD, showing that individuals with TMJD exhibit impaired cranio‐cervico‐mandibular function, postural stability, and proprioception, which may be influenced by pain severity and altered scapulothoracic mechanics [10, 11]. No research has explored this relationship in the Iranian population, highlighting the need for such a study. This study investigates the connection between TMJD and head–neck posture in patients at Tehran University’s Faculty of Dentistry by measuring the craniohorizontal angle (CHA) angle (the angle formed between an imaginary horizontal line across the face and the line extending from the tragus to the outer corner of the eye) and craniovertebral angle (CVA) angle (the angle formed between the horizontal line and the line drawn from the C7 vertebra to the tragus). A reduced CVA, indicating forward head posture, is clinically significant, as it is associated with increased cervical muscle strain, pain, and functional limitations, which may exacerbate or perpetuate TMJD symptoms.

## 2. Materials and Methods

This case–control study aimed to explore the relationship between TMJD and head and neck posture in patients visiting the maxillofacial surgery department at Tehran University of Medical Sciences in 2019. This study was approved by the Institutional Ethics Committee of Tehran University of Medical Sciences (IR.TUMS.DENTISTRY.REC.1398.097).

A prospective power analysis was conducted using PASS 11 software. Based on data from Faulin et al. [12] showing a mean CVA difference of 2.0° between groups with a standard deviation of 5.64°, we determined that 65 participants per group (total *N* = 130) would provide 80% power (*β* = 0.20) to detect significant differences using a two‐tailed *t*‐test (*α* = 0.05). The study involved two groups: a patient group consisting of individuals seeking treatment for TMJD and a control group of healthy university employees. All participants were aged between 18 and 50 years and provided voluntary informed consent.

Patients were diagnosed with TMJD according to the research diagnostic criteria for temporomandibular disorders (RDC/TMD) and a positive compression test. To be included in the patient group, individuals were required to have experienced pain in the TMJ or masticatory muscles for a minimum of 3 months, unrelated to inflammation, infection, trauma, or cancer. Additionally, the pain intensity had to be rated as at least 3 out of 10 on a visual analog scale (VAS). This threshold was chosen because pain of ≥3 is generally regarded as clinically significant and likely to interfere with daily functioning [13]. Furthermore, this criterion is consistent with the RDC/TMJD diagnostic guidelines for persistent TMJD‐related pain [14].

The study excluded individuals with articular disc disorders, pre‐existing pathological conditions, prior surgeries, restricted neck or jaw mobility, or other musculoskeletal or systemic disorders. Additional exclusion criteria included the use of medications that affect the musculoskeletal system or the presence of orthodontic appliances. The healthy group consisted of individuals who were free from jaw or neck pain or TMJD symptoms for at least 1 year. They were recruited through announcements, provided informed consent, and had their confidentiality protected.

### 2.1. Photographic Protocol

Lateral photographs were taken using a standardized protocol. A digital camera (Model: Canon EOS 90D, lens: 50 mm) was mounted on a tripod 2 m from the participant at tragus height. Lighting was controlled to minimize shadows. A plumb line in the background provided a vertical reference. Participants stood barefoot with their median sagittal plane aligned to a ground marker, teeth occluded, and eyes focused on a vertical line.

A camera, positioned 2 m away at tragus height, captured two lateral photos once the subjects achieved a “Natural Head Position” by focusing on a distant point and taking deep breaths. Markers on the C7 vertebra aided posture analysis, and photos were assessed for head and neck posture angles.

Using Photoshop, CHA and CVA angles were measured as averages of two photos. CHA is the angle from the eye corner to tragus to the horizontal line, and CVA is from the C7 marker to tragus to the horizontal line. The horizontal line passes through the tragus center and is perpendicular to the wall’s vertical line.

### 2.2. Blinding

**Figure 1:**
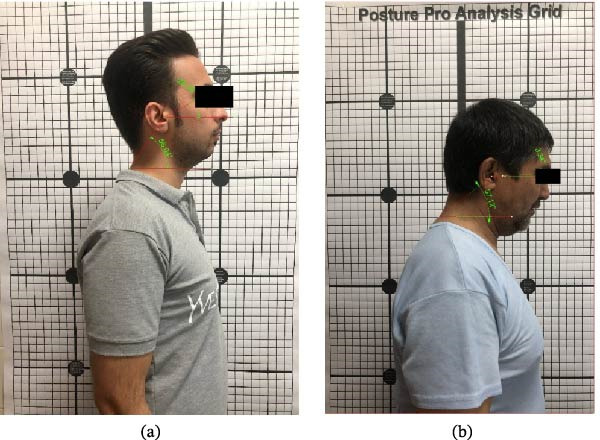
Comparison of CHA and CVA angles between a healthy individual (a) and a TMD patient (b). The CHA angle is defined as eye corner–center of tragus—horizontal line, while the CVA angle is tip of C7 marker–center of tragus—horizontal line. The horizontal line passes through the center of the tragus and is perpendicular to the vertical line of the wall opposite the camera. Published with patient consent.

The assessor measuring the CHA and CVA angles was blinded to group allocation. All photographs were anonymized and assigned random codes before analysis to prevent identification of case or control status. Figure 1 compares CHA and CVA angles in a healthy patient (a) and a TMJD patient (b).

### 2.3. Participant Flow

**Figure 2:**
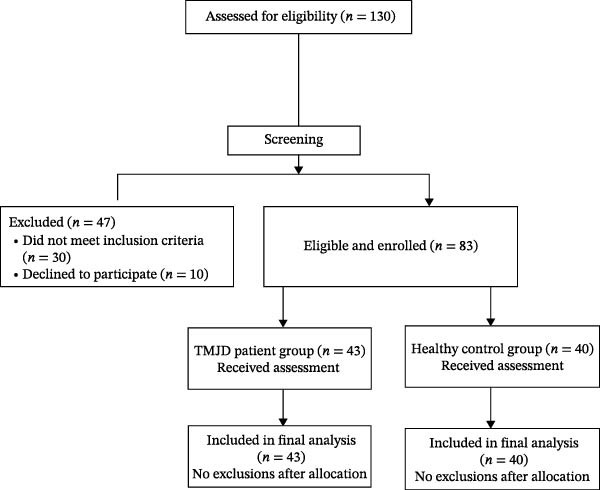
Participant flow diagram.

After initial screening of 130 potential participants (65 per group), a final sample of 43 TMJD patients and 40 healthy controls met all inclusion/exclusion criteria and had complete photographic data for analysis. The participant flow is detailed in Figure 2.

Data were analyzed using SPSS Version 21, with a significance set at *p*  < 0.05. Quantitative data were summarized by mean and standard deviation and qualitative data by frequency. Normality was checked with Kolmogorov–Smirnov. Pearson or Spearman tests evaluated correlations, chi‐square tests nominal relationships, and independent *t*‐tests or ANOVA were applied if parametric conditions were met; otherwise, equivalent nonparametric tests were used.

## 3. Results

This study was carried out to investigate how TMJ dysfunction relates to head and neck posture in patients undergoing treatment at the maxillofacial surgery department at the Dentistry Faculty of Tehran University of Medical Sciences.

### 3.1. Participant Characteristics

The final sample included 43 TMJD patients (mean age 38.13 ± 9.30 years) and 40 controls (mean age 25.50 ± 1.64 years). Demographic and clinical characteristics are presented in Table 1. Included are demographic details, such as gender and age, as well as clinical indicators like jaw deviation, TMJ clicking, neck pain, bruxism, clenching, pain while mouth opening and tenderness in various muscles.

**Table 1:** Participant demographic and clinical characteristics.

Variable	Control group	Case group
Gender (N)		
Women	21	28
Men	19	15
Average age (years)	25.5 ± 1.64	38.13 ± 9.30
Jaw deviation present	0%	62.80%
Jaw deviation absent	100%	37.20%
TMJ click present	0%	74.40%
TMJ click absent	100%	25.60%
Masseter muscle tenderness present	0%	86.00%
Masseter muscle tenderness absent	100%	14.00%
Temporal muscle tenderness present	0%	81.40%
Temporal muscle tenderness absent	100%	18.60%
Medial pterygoid muscle tenderness present	0%	90.70%
Medial pterygoid muscle tenderness absent	100%	9.30%
Lateral pterygoid muscle tenderness present	0%	83.70%
Lateral pterygoid muscle tenderness absent	100%	16.30%
Pain while mouth opening present	0%	83.70%
Pain while mouth opening absent	100%	16.30%
Neck pain present	0%	79.10%
Neck pain absent	100%	20.90%
Bruxism present	0%	86.00%
Bruxism absent	100%	14.00%
History of trauma present	0%	65.10%
History of trauma absent	100%	34.90%
Clenching present	0%	65.10%
Clenching absent	100%	34.90%

### 3.2. Postural Angles

**Figure 3:**
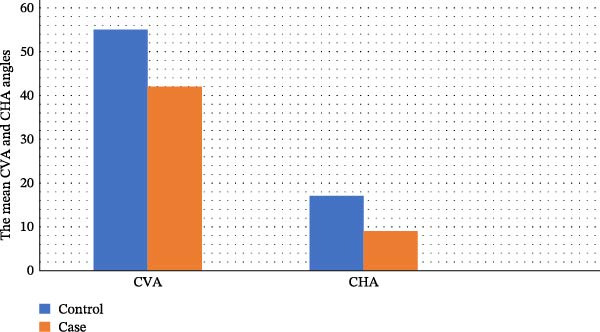
Analysis of the average CVA and CHA Angles in two groups.

The TMJD group had a significantly lower mean CVA (41.1° ± 6.61°, 95% CI [38.9, 43.3]) compared to the control group (56.83° ± 4.33°, 95% CI [55.4, 58.2]), with a mean difference of −15.73° (95% CI [−19.1, −12.3], *p* = 0.027). These results indicate a difference in the average CHA and CVA angles between the two groups. (Figure 3) In the comparison of the mean of CVA angle between the two groups, statistically significant differences were observed (*p* = 0.027). The difference in mean CHA was not statistically significant (TMJD: 9.67° ± 6.98°; Control: 15.91° ± 3.34°; *p* = 0.091). (Table 2)

**Table 2:** Comparison of Craniohorizontal (CHA) and Craniovertebral (CVA) angles between TMJD and control groups.

Variable	Mean ± SD		^a^ *p*‐Value
	Case	Control	
CHA	9.67° ± 6.98°	15.91° ± 3.34°	0.091
CVA	41.1° ± 6.61°	56.83° ± 4.33°	0.027

^a^Independent samples *T*‐test: (*p*‐value: significant <0.05).

### 3.3. Regression Analysis

Multiple linear regression (Table 3) revealed that a lower CVA was significantly associated with the presence of jaw deviation (*β* = −4.05, *p* = 0.021), clicking (*β* = −5.93, *p* ≤ 0.001), bruxism (*β* = −8.27, *p* = 0.009), clenching (*β* = −3.18, *p* = 0.045), and older age (*β* = −0.46, *p* ≤ 0.001), accounting for 77.8% of its variance (*R* ^2^ = 0.778, *p* ≤ 0.001).

**Table 3:** Multiple linear regression analysis of predictors for Craniovertebral Angle (CVA).

Variable	Beta coefficient	Standard error	*p*‐Value
Deviation	−4.045	1.71	0.021
Clicking	−5.932	1.46	≤0.001
Neck pain	5.639	3.00	0.064
Bruxism	−8.273	3.07	0.009
Clenching	−3.176	1.561	0.045
Age	−0.456	0.06	≤0.001

## 4. Discussion

TMJD describes orofacial issues caused by the TMJ and its surrounding structures [15]. Headache is reportedly the most common sign of TMJD. The second most prevalent symptom is clicking [16]. However, in our study, we found pain in the medial pterygoid muscle and teeth grinding to be the most frequently observed symptoms. Although TMJD is a common condition, studies have reported varying prevalence rates due to variations in measurement criteria and techniques.

We aimed to determine whether TMJD patients exhibited different head and neck positions and postures compared with healthy subjects. We measured certain craniocervical and facial angles, including the CVA and reference points like the C7 vertebra and tragus of the ear, to determine the relationship between posture and TMJD [17]. The C7 vertebra and tragus of the ear showed consistent results in determining forward head position without being significantly influenced by segmental distance or individual postural variations.

The outcomes of our study demonstrate a statistically significant correlation between TMJD and head and neck posture. Specifically, patients with TMJD exhibited decreased CHA and CVA angles compared to control subjects. In the present study, the control group demonstrated a CHA with a mean of 15.91°, which reflects a normative facial tilt [18]. Furthermore, their CVA, with a mean of 56.83°, exceeds the threshold of 53° established for a neutral head posture [19]. These values are consistent with a healthy postural profile, supporting the representativeness of our control group. Regression analysis of significant predictors associated with the CVA angle, as shown in Table 3, reveals a significant negative correlation between the criteria, including bruxism, clenching, and age.

The finding of a significantly reduced CVA in TMJD patients (mean difference >15°) is clinically meaningful. A forward head posture increases mechanical strain on the craniocervical and masticatory musculature, potentially contributing to pain and dysfunction. This supports the inclusion of postural evaluation in TMJD assessment.

Parafunctional habits like bruxism and clenching lead to heavier loads in the TMJ and condylar fossa, which in turn causes degeneration of the joint and condyle and even mandibular deviation. As Santos et al. [20] concluded in their 2020 study, these forces will result in irreversible deformities in jaw structure and decreased height in the middle third of the face, reducing CHA angle. Additionally, the CHA angle is negatively correlated with age. One possible explanation for this could be that the jaw’s position is affected as a result of the elasticity of the muscles and supporting tissue surrounding the TMJ decreasing with age. Li et al. [21] also found that aging negatively affects the elasticity of the tissue surrounding the jaw and alters its general structure.

As indicated in Table 3, deviation, clicking, bruxism, and age correlate negatively with CVA angle due to compensatory mechanisms in head positioning. Aging is associated with degenerative changes in musculoskeletal structures, including the TMJ and cervical spine. These changes can reduce flexibility and alter posture, decreasing CVA angle [21]. Although neck pain was not retained as a statistically significant predictor in our final model, it is a common comorbidity in TMJD. It may have clinical relevance that was not captured in our analysis. Neck pain leads to compensational changes in head and neck position, indirectly affecting the CVA angle [22].

Our finding of a reduced CVA in TMJD patients aligns with emerging research indicating that TMJD is associated with broader postural control deficits and altered proprioception within the cranio‐cervico‐mandibular chain [10]. This suggests that the observed forward head posture may be one component of a more systemic sensorimotor dysfunction.

Saito et al. [17] observed a close relationship between body posture and TMJD. However, it is yet to be determined which is the cause and which is the result. Regardless, posture assessment can aid in preventing, diagnosing, and even treating TMJDs [17].

Motta and colleagues conducted a study on the relation between cervical posture and TMJD in adolescents. The researchers observed that patients suffering from mild to moderate TMJD had a more forward head position. One possible reason for this could be that a more forward head position causes more tension in masticatory muscles, which may worsen TMJD. It was also observed that patients with mild to moderate TMJD had a more forward head position compared with patients suffering from severe TMJD [23].

Armijo‐Olivo et al. [24] found that TMJD patients exhibited a more forward head position and a statistically significant increase in the CHA angle.

Nevertheless, it should be noted that postural angles, on their own, did not significantly correlate with neck issues, jaw issues, or pain intensity when factors such as age, height, and weight were controlled. According to the regression analysis, postural variables were accountable for only a small percentage of the variability in these outcomes; thus, other factors are likely to be responsible for jaw and neck concerns. This result supports Falla et al.’s study [25], which stated that changes in head position were not correlated with either reduced pain or disability. It implies that the relationship between pain and dysfunction transcends postural alignment. However, in our study, clenching, bruxism, and age are the factors that influence the reduction of CVA. It can be concluded that these factors have a considerable impact on ergonomics in TMJD patients.

The clinical implication of our findings, supported by recent interventional research, is that TMJD management may benefit from extending beyond the orofacial region. For instance, exercises targeting the scapulothoracic region have been shown to improve proprioception and postural stability in individuals with cranio‐cervico‐mandibular malalignment [11]. This supports the integration of postural assessment and targeted physiotherapy into a multidisciplinary treatment approach.

Our research utilized photogrammetry and anatomical surface markers to obtain reliable measurements of postural angles, although radiographic evaluations are generally considered more accurate than this method. Future studies should consider both imaging and multifactorial models to comprehensively understand the causes of pain and disability associated with TMJ disorders.

## 5. Conclusion

Our findings reveal significant postural differences, most notably in the CVA angle, between the TMJD and control groups. Notable variations were observed in the angle of the CVA between the two groups, highlighting a connection between age, clenching, bruxism, and a reduction in this angle, which correlates with poor head and neck posture. This suggests that age, clenching, and bruxism are critical factors influencing postural alignment in individuals with TMJD, warranting further attention in clinical assessments and interventions for posturally impaired TMJD patients.

## 6. Limitations

This study has several limitations. The significant age difference between groups, despite being a key predictor in the regression, is a potential confounder. Our reliance on surface photogrammetry, while noninvasive, lacks the bony detail provided by radiographic imaging (e.g., lateral cephalometry). Furthermore, unmeasured confounders such as occupational ergonomics, screen time, and psychological stress were not assessed, which may influence both posture and TMJD symptoms. Generalizability may be limited to similar clinical populations.

## 7. Recommendations

Prospective longitudinal studies are needed to establish causality. Interventional trials should examine whether physiotherapy targeting postural correction or specific management of bruxism improves TMJD outcomes. Combining photogrammetry with advanced imaging could offer more comprehensive insights.

## Author Contributions

Amir Jalal Abbasi designed the study, developed the methodology, and oversaw the manuscript formatting. Mona Mohajeri Tehrani was involved in data collection, analysis, supervision, reviewing, and editing and submission of the final draft. Abbas Karimi and Mahsa Sadat Seyedi Konjani were involved in validation of the data and writing the original draft. Shamim Chinian was involved in data collection and also in reviewing and editing of the final draft.

## Funding

This work was self‐funded.

## Disclosure

All authors reviewed the final manuscript and approved it for submission, agreeing to be accountable for all aspects of the work.

## Ethics Statement

Mona Mohajeri Tehrani hereby declare that all ethical standards have been adhered to in the preparation of the submitted article.

## Conflicts of Interest

The authors declare no conflicts of interest.

## Data Availability

The data that support the findings of this study are available from the corresponding author upon reasonable request.
